# The effect of two clinical criteria in the assessment of caries lesions around restorations in children (CARDEC-03): study protocol for a diagnostic randomized clinical trial

**DOI:** 10.12688/f1000research.23801.3

**Published:** 2021-01-11

**Authors:** Bruna Lorena Pereira Moro, Cácia Signori, Raiza Dias Freitas, Laura Regina Antunes Pontes, Tathiane Larissa Lenzi, Tamara Kerber Tedesco, Daniela Prócida Raggio, Mariana Minatel Braga, Kim Rud Ekstrand, Maximiliano Sérgio Cenci, Fausto Medeiros Mendes, CARDEC collaborative group, CaCIA collaborative group

**Affiliations:** 1 Department of Pediatric Dentistry, School of Dentistry, University of São Paulo, São Paulo, Brazil; 2 Graduate Program in Dentistry, Federal University of Pelotas, Pelotas, Rio Grande do Sul, Brazil; 3 Department of Surgery and Orthopedics, School of Dentistry, Federal University of Rio Grande do Sul, Porto Alegre, Brazil; 4 Graduate Program in Dentistry, Ibirapuera University, São Paulo, Brazil; 5 Department of Odontology, Faculty of Health and Medical Sciences, University of Copenhagen, Copenhagen, Denmark

**Keywords:** Randomized Controlled Trial, Dental Caries, Diagnosis, Permanent Dental Restoration, Dental Restoration Repair, Pediatric Dentistry

## Abstract

**Introduction: **The detection of caries lesions around restoration can be challenging. Therefore, the use of some criteria has been proposed in order to give more objectivity to the diagnosis process. Two of them are the International Dental Federation (FDI) and the Caries Associated with Restorations and Sealants (CARS) criteria. Both methods have a different approach to caries, and it is not possible to know which one of them is the best to use in clinical practice to assess restorations in children. Thus, the present protocol aims to evaluate the effect of the use of the FDI and CARS criteria in the assessment of caries lesions around restorations in primary teeth on outcomes related to oral health in children and costs resulting from the assessments.

**Methods and analysis:** A total of 626 restorations of children from three to 10 years were randomly assessed and are being treated following the FDI criteria (FDI group) or CARS criteria (CARS group). Participants will be followed-up after six, 12, 18, and 24 months. The primary outcome will be the need for a new intervention in the evaluated restorations. This outcome consists of several components, and each of these events will be analyzed separately as secondary outcomes. The changes in children’s oral health-related quality of life and the cost of the restoration dental treatments will also be analyzed as secondary outcomes. The methods will be compared using the Cox regression model with shared frailty. A significance level of 5% will be adopted for all statistical analyses.

**Discussion: **This will be the first randomized clinical study carried out regarding the detection of caries lesions around restorations in primary teeth.

**Trial registration:** The study underwent registration in Clinicaltrials.gov ( NCT03520309) on 9 May 2018.

## Introduction 

Caries lesions around restoration, also known as secondary caries or recurrent caries, are the main reason for restoration failure ^1^. However, the detection of these lesions can be challenging for a few reasons, as the presence of gaps between the restoration and tooth surface ^2^ and the presence of stained margins on resin-based composite restorations makes it difficult to differentiate between lesions and demineralization ^3^. For this reason, the use of some criteria has been proposed to give more objectivity to the diagnosis process.

 One such set of criteria is the International Dental Federation (FDI) criteria ^4^, developed in 2007. Although largely used to assess restorations, it evaluates some aspects that might not be directly related to caries lesions, such as marginal staining and marginal adaptation. However, these aspects could be relevant to be evaluated when using the FDI criteria since many dentists and studies associate marginal staining and defects in the marginal adaptation with the presence of caries lesion around the restoration ^5^. Using these criteria may lead to a more interventional approach. Another set of criteria is the Caries Associated with Restorations and Sealants (CARS) criteria, which has been integrated into the International Caries Classification and Management System ^6^ and its more recent update, named CariesCare 4D ^7^. The CARS criteria ^6^ focus on aspects related to caries and not on other possible reasons for restoration failure. This method is probably more conservative when it comes to restoration reintervention.

 When it comes to the management of restorations in primary dentition, it is not possible to know if a more conservative or invasive approach would bring more benefits to children. Restorations that are repaired seem to be more likely to have an additional treatment compared to restorations that are replaced ^8^. On the other hand, replacement often causes the loss of healthy dental structure ^9, 10^, leading to a repeated restorative cycle ^11^, increasing the professional time and costs for health systems ^9^.

It would be preferable that the criteria for assessing caries around restorations in children is in line with the philosophy of minimal intervention dentistry ^12^. However, the majority of studies about the detection of these lesions were performed *in vitro*, assessed caries lesion in permanent teeth, and did not evaluate relevant aspects to the clinical practice ^5, 13^. This lack of evidence inspires the conduction of a third study, which is part of an initiative that aims to build scientific evidence for diagnostic strategies in children - CARies DEtection in Children n° 3 (CARDEC-03).

Thus, this trial aims to evaluate the effect of the use of two different visual criteria, the FDI and CARS criteria, for assessing caries lesions around restorations in primary teeth on outcomes related to children’s oral health and costs resulting from the assessments. We hypothesize that the diagnostic criteria that lead to a more conservative approach would bring more benefits to children’s oral health, decreasing the treatment costs and professional time.

## Methods

A controlled, triple-blind (participant, care provider, outcomes assessor), randomized clinical trial with two parallels arms (1:1) is being carried out. The present protocol is reported according to the Standard Protocol Items: Recommendations for Interventional Trials (SPIRIT) guidelines ^14^. The completed checklist can be accessed on Figshare ^15^.

 The local ethics committee from the School of Dentistry of the University of São Paulo, São Paulo, Brazil, previously approved the study (registration no. 2.291.642) on 22 September 2017. The participants of the study were recruited from 16 November 2017 to 30 November 2018. The trial was retrospectively registered on Clinicaltrials.gov ( NCT03520309) on 9 May 2018 due to of a lack of awareness that registration must occur before enrollment begins. No changes were made to the study after approval by the local ethics committee in 2017, and no results were analyzed before the trial registration on Clinicaltrials.gov. The authors are aware of possible causes of publication bias and selective reporting, and are committed to promoting complete transparency in our research.

## Participants, interventions, and outcomes

### Study setting

This trial is being conducted at the School of Dentistry Dental Clinic of the University of São Paulo, Brazil. The participants (3 to 10 years old) were selected from a list of patients who sought dental treatment at the School of Dentistry. Those that fulfilled the eligibility criteria were randomly allocated to the intervention groups. A random sequence was generated using the website “ Sealed Envelope” through the tool “Create a randomisation list”. The patients were included in the study after their legal guardians signed the informed consent form and literate children signed an assent form. Both documents are available as *Extended data* in English ^16, 17^ and the original language ^18, 19^.

### Participant eligibility

The inclusion criteria for the present study are children:

a) Who have sought treatment at the School of Dentistry;
b) From three to 10 years-old;
c) Presenting at least one restoration of any restorative material (composite resin, amalgam or glass ionomer cement), regardless of its condition, on a primary tooth (anterior or posterior) without fistula, abscess, pulp exposure, history of spontaneous dental pain or mobility.

The exclusion criteria for the present study are children:

a) Whose parents refuse to participate in the study;
b) Who did not agree to participate, or showed behavior problems during the first appointment.

All children’s restorations which fulfill the inclusion criteria were included for the assessment.

### Allocation: sequence generation and concealment mechanism

Firstly, participants were stratified into different strata: (1) children aged 3 to 6 years presenting three restorations or less; (2) children aged 7 to 10 years presenting three restorations or less; (3) children aged 3 to 6 years presenting more than three restorations; (4) children aged 7 to 10 years presenting more than three restorations. The number of restorations considered for stratification was those placed in primary and permanent teeth. Then, randomization using blocks of different sizes (2, 4, 6 or 8) was performed within each stratum.

All participants of the study could be classified as having a high caries risk since past caries experience is the most important component for the development of caries lesions ^20^. However, stratified randomization was performed considering the children number of restorations to subdivide them in children with higher and lower caries experience. The caries lesion activity was not considered for randomization. On the other hand, the children's age was a parameter for stratification in order to consider the different time of exfoliation of the evaluated teeth. In this way, the number of teeth with different time of exfoliation was balanced between the FDI and CARS criteria.

The random sequence was generated using the website “Sealed Envelope” through the tool “Create a randomisation list”. It was done by an external examiner and to guarantee allocation confidentiality, blocks with allocation sequences were kept in opaque sequential envelopes.

### Interventions

A preliminary visual inspection was performed to assess all participants’ dental surfaces according to the International Caries Detection and Assessment System (ICDAS) ^21^ described in the CariesCare 4D to detect and assess the caries lesions stage and activity ^7^. The assessment was performed by an examiner (LRAP) who is not participating in the subsequent phases of the study. All the assessments of the study are being conducted under a dental clinic setting using a dental chair and artificial illumination. Participants’ teeth receive a professional oral hygiene using a rotating bristle brush, pumice/water slurry and dental floss. A plane buccal mirror and a ball-point probe are being used for all visual inspection and tactile examination of the clinical trial.

Then, children meeting the inclusion criteria were classified into subgroups for further block stratification, according to the number of restorations present in mouth (0 to 3 restorations vs. more than three restorations) and age (3 to 6 years old vs. 7 to 10 years old).

**Figure 1.**
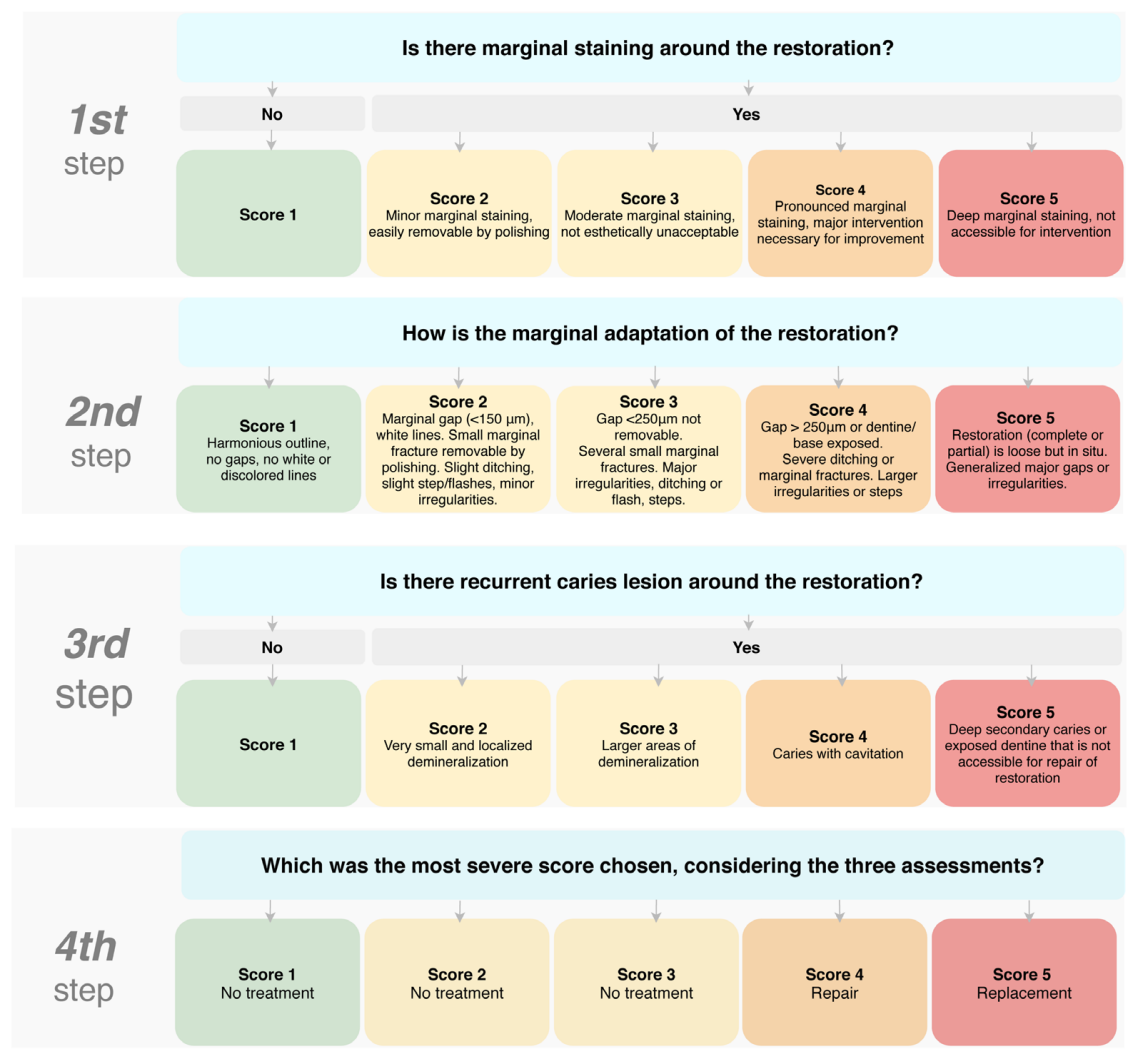
Patient’s plan decision flowchart based on the International Dental Federation (FDI) criteria.

**Figure 2.**
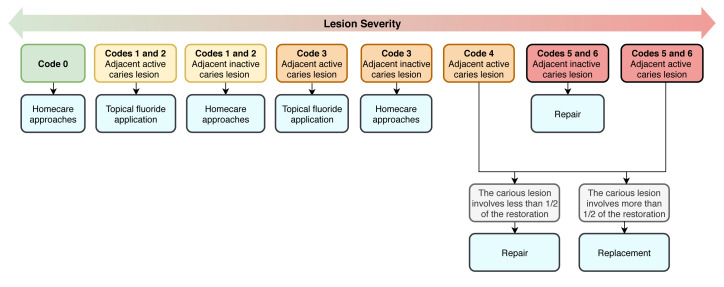
Patient’s plan decision flowchart based on the Caries Associated with Restoration and Sealants (CARS) criteria.

The children included in the study were randomly allocated in two groups to have their restorations evaluated and treated according to different clinical criteria for caries lesion around restoration:

a) FDI group: diagnosis and treatment decision based on the International Dental Federation (FDI) criteria ^4^ ( Table 1 and Figure 1).
b) CARS group: diagnosis according to the Caries Associated with Restorations and Sealants (CARS) detection criteria, described in the ICCMS ^6^ and in CariesCare 4D ^7^ ( Table 2 and Figure 2), and proposed treatment decision ( Table 3). The definitions and characteristics of activity for primary caries from CariesCare International 4D will also be used in association ( Table 4).

**Table 1.** International Dental Federation (FDI) criteria linked to the treatment decision.

FDI scores					FDI treatment
Scores	Classification	Marginal staining*	Marginal adaptation	Recurrence of caries	Indication
**1**	**Clinically excellent/** **very good**	No marginal staining.	Harmonious outline, no gaps, no white or discolored lines	No secondary or primary caries	No treatment
**2**	**Clinically good**	Minor marginal staining, easily removable by polishing.	Marginal gap (<150 μm), white lines. Small marginal fracture removable by polishing. Slight ditching, slight step/flashes, minor irregularities.	Very small and localized demineralization	No treatment
**3**	**Clinically sufficient/** **satisfactory**	Moderate marginal staining, not esthetically unacceptable.	Gap <250μm not removable. Several small marginal fractures. Major irregularities, ditching or flash, steps.	Larger areas of demineralization	No treatment
**4**	**Clinically** **unsatisfactory**	Pronounced marginal staining; major intervention necessary for improvement.	Gap > 250μm or dentine/ base exposed. Severe ditching or marginal fractures. Larger irregularities or steps.	Caries with cavitation	Repair
**5**	**Clinically poor**	Deep marginal staining, not accessible for intervention.	Restoration (complete or partial) is loose but in situ. Generalized major gaps or irregularities.	Deep secondary caries or exposed dentine that is not accessible for repair of restoration.	Replacement

This table was created based on information from Hickel *et al.* 2010 ^4^.

**Table 2.** Caries Associated with Restorations and Sealants (CARS) criteria.

Caries Associated with Restorations and Sealants codes		
**Code 0**	**Sound tooth surface with** **restoration or sealant**	A sound tooth surface adjacent to a restoration/sealant margin. There should be no evidence of caries (either no or questionable change in enamel translucency after prolonged air drying for 5 seconds). Surfaces with marginal defects less than 0.5mm in width (i.e. will not admit the ball end of the CPI Probe), developmental defects such as enamel hypoplasias; fluorosis; tooth wear (attrition, abrasion and erosion), and extrinsic or intrinsic stains will be recorded as sound. Stained margins consistent with non-carious habits (e.g. frequent tea drinking) and which do not exhibit signs consistent with demineralization should be scored as sound.
**Code 1**	**First visual change in enamel**	When seen wet there is no evidence of any change in color attributable to carious activity, but after prolonged air drying (for approximately 5 seconds) an opacity or discoloration consistent with demineralization is visible that is not consistent with the clinical appearance of sound enamel.
**Code 2**	**Distinct visual change in enamel/** **dentin adjacent to a restoration** **margin**	If the restoration margin is placed on enamel the tooth must be viewed wet. When wet there is an opacity consistent with demineralization or discoloration that is not consistent with the clinical appearance of sound enamel (Note: the lesion is still visible when dry). If the restoration margin is placed on dentin: Code 2 applies to discoloration that is not consistent with the clinical appearance of sound dentin or cementum.
**Code 3**	**Carious defects of <0.5 mm with** **the signs of code 2**	Cavitation at the margin of the restoration/sealant less than 0.5mm, in addition to either an opacity or discoloration consistent with demineralization that is not consistent with the clinical appearance of sound enamel or with a shadow of discolored dentin.
**Code 4**	**Marginal caries in enamel/dentin/** **cementum adjacent to restoration** **with underlying dark shadow** **from dentin**	The tooth surface may have characteristics of code 2 and has a shadow of discolored dentin which is visible through an apparently intact enamel surface or with localized breakdown in enamel but no visible dentin. This appearance is often seen more easily when the tooth is wet and is a darkening and intrinsic shadow which may be grey, blue, orange, or brown in color. Note: view tooth wet and then dry. This lesion should be distinguished from amalgam shadows.
**Code 5**	**Distinct cavity adjacent to** **restoration**	Distinct cavity adjacent to restoration/sealant with visible dentin in the interfacial space with signs of caries as described in code 4, in addition to a gap > 0.5mm in width. OR In those instances where margins are not visible, there is evidence of discontinuity at the margin of the restoration/sealant and tooth substance of the dentin as detected by 0.5mm ball-ended probe run along the restoration/sealant margin.
**Code 6**	**Extensive distinct cavity with** **visible dentin**	Obvious loss of tooth structure, the extensive cavity may be deep or wide and dentin is clearly visible on both the walls and at the base.

This table was created based on information from Pitts *et al.* 2016 ^6^ and Martignon *et al.* 2019 ^7^.

**Table 3.** Treatment decision linked to the Caries Associated with Restoration and Sealants (CARS) criteria.

CARS code	CARS Treatment		
**0**	**No treatment**	No treatment	**-**
**1**	**Non-operative** **treatment**	No treatment ^1^ Topical fluoride application ^2^	^1^ Adjacent inactive lesion ^2^ Adjacent active lesion.
**2**		No treatment ^1^ Topical fluoride application ^2^	
**3**		No treatment ^1^ Topical fluoride application ^2^	
**4**	**Operative treatment**	Repair Replacement ^3^	^3^ Replacement should be indicated in case the carious lesion involves more than half of the restoration.
**5**		Repair Replacement ^3^	
**6**		Repair Replacement ^3^	

This table was created based on information from Pitts *et al.* 2016 ^6^ and Martignon *et al.* 2019 ^7^.

**Table 4.** Characteristics of active and inactive caries linked to caries around restorations system - Caries Associated with Restoration and Sealants (CARS) - adapted.

ICCMS Code	Characteristics of Lesion	
	Signs of Active Lesions	Signs of Inactive Lesions
ICCMS Initial and Moderate Caries Stage	Surface of enamel is whitish/yellowish; opaque with loss of luster, feels rough when the tip of the ball- ended probe is moved gently across the surface. Lesion is in a plaque stagnation area, i.e. in the entrance of pits and fissures, near the gingival margin or, for proximal surfaces, below or above the contact point. The lesion may be covered by thick plaque prior to cleaning.	Surface of enamel is whitish, brownish or black. Enamel may be shiny and feels hard and smooth when the tip of the ball-ended probe is moved gently across the surface. For smooth surfaces, the caries lesion is typically located at some distance from the gingival margin. Lesion may not be covered by thick plaque prior to cleaning.
ICCMS Extensive Caries Stage	Dentine feels soft or leathery on gentle probing.	Dentine is shiny and hard on gentle probing.

This table was created based on information from Pitts *et al.* 2016 ^6^ and Martignon *et al.* 2019 ^7^.

**Figure 3.**
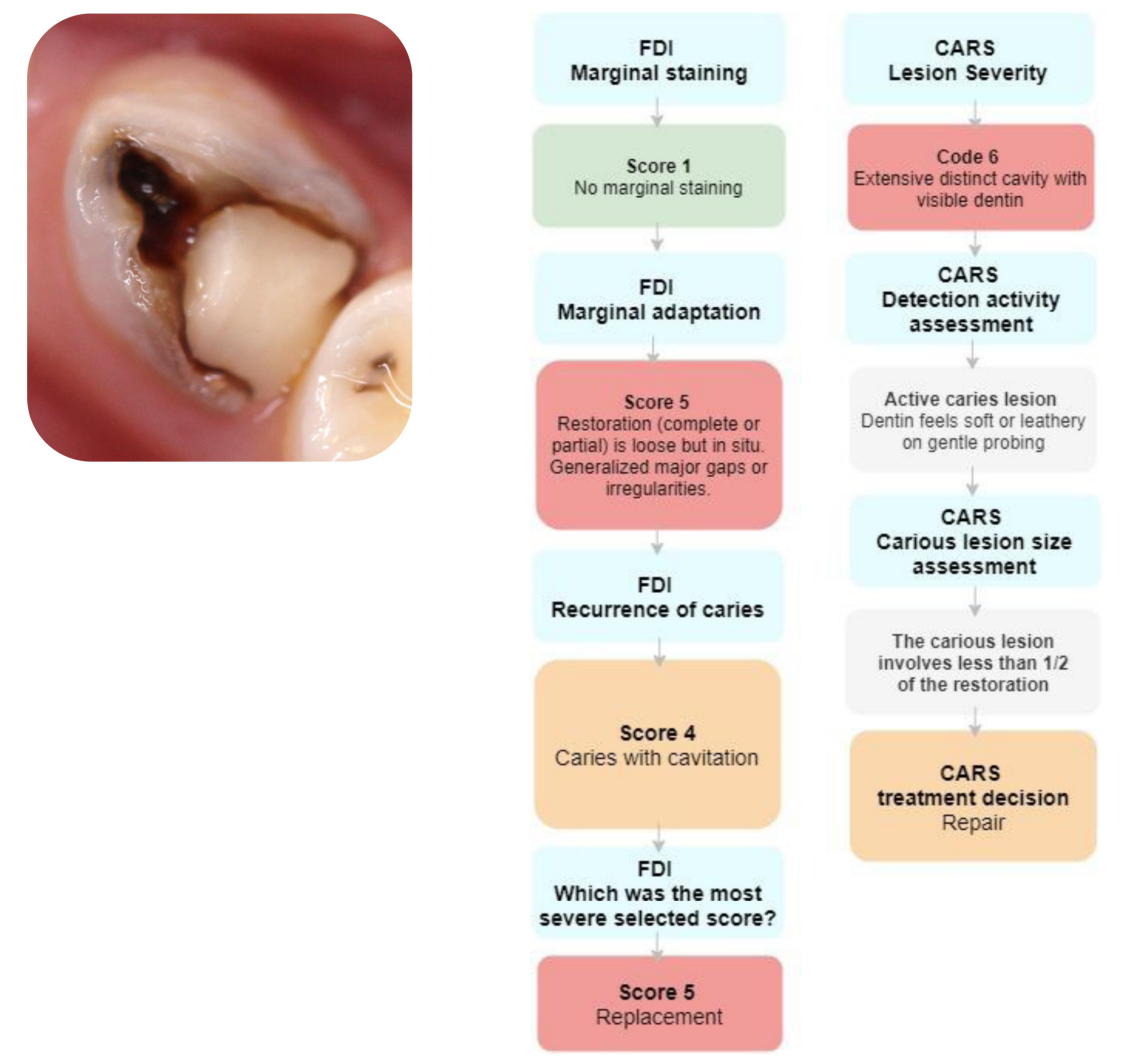
Clinical example of the restoration assessment performed in a primary posterior tooth according to the FDI and CARS criteria.

A clinical example of the restoration assessment performed with both FDI and CARS criteria is illustrated in Figure 3.

The restorations assessment was performed by an examiner (BLPM), who was trained and calibrated before the beginning of the study. Calibration involves a lecture of clinical criteria, and training was carried out using photos of clinical cases. The web-based training and calibration tool ICDAS Calibration for ICCMS(TM) by ICCMS e-learning was used for this purpose.

After these procedures, the examiner evaluated restorations in 10 children who did not participate in the clinical trial. The examiner repeated the same evaluation, in the same 10 children, for intra-examiner agreement. A benchmark examiner (TLL) also performed the tests to assess inter-examiner reproducibility twice in the same sample of children. In this way, the exams were compounded, and the weighted kappa scores were re-calculated. The assessment of children included in the study started after the intra-examiner and inter-examiner weighted kappa value reached values greater than 0.75 for both FDI and CARS criteria.

For examinations using the FDI criteria, all tooth surfaces are dried before. When using the CARS criteria, teeth are examined firstly wet and then dried for 5 seconds with a dental 3-in-1 air water syringe.

The first assessment was performed with the participant’s allocated group (FDI or CARS). After reaching the diagnosis and treatment decision according to the allocated group, the same examiner performed a second assessment according to the other criteria. This procedure aims to compare the methods since a cross-sectional study was developed nested in this randomized clinical trial. The second assessment did not influence or change the classification and treatment decision proposed by the criteria the participant is allocated. If a legal guardian presents a complaint related to any children’s restoration, it can be repaired or replaced independently of the criteria used. The scores obtained with the restoration assessment were collected using a specific sheet that can be found as *Extended data* in English ^22^ and Portuguese ^23^.

At the first appointment, legal guardians were asked to answer a questionnaire to assess the impact on children’s oral health-related quality of life. The instrument used was the Brazilian version ^24, 25^ of the Early Childhood Oral Health Impact Scale (ECOHIS) ^26^. We decided to use an instrument answered by the parents since our sample's age range is large and involves small children who would have difficulty answering other questionnaires. This choice was made to allow data to be collected for the entire sample and for the same instrument to be standardized. Besides that, an anamnesis related to children’s health and medical history was carried out (this form is available as *Extended data* in English ^27^ and original language ^28^). At the end of the first appointment, oral hygiene instructions were delivered, showing the correct use of toothbrush and fluoride toothpaste (1000 to 1500 ppm of fluoride) ^29^. Dietary advice was also given to all participants and their parents or legal guardians to reduced intake of free sugars throughout the life course ^30^.

For all appointments, the time spent and materials used on patient care are collected using a specific sheet that can be found as *Extended data* in English ^31^ and original language ^32^. Parents or guardians are asked about transportation and absenteeism in the workplace.

### Dental treatment protocols 

In the subsequent appointments, dental treatments following a predefined protocol are being performed by postgraduate dental students in Pediatric Dentistry, who are blind to the criteria used to reach the treatment decision. In all situations, if there is active dentine tissue, it is removed using dentin excavators. Diamond burs are used to remove the restorations, if necessary.

The treatment decisions for the restorations evaluated according to the FDI and CARS criteria are being classified into:

- No treatment: no intervention needed and the restoration will be followed-up;
- Professional topical fluoride application: a treatment for non-cavitated active caries lesions detected by the CARS criteria;
- Refurbishment: restorations finishing and polishing;
- Repair: minimally invasive approach resulting in the addition of a restorative material, with or without a preparation of the restoration and/or dental hard tissues ^33^. Composite resin or glass ionomer cement will be used as a restorative material;
- Replacement: complete removal of the restoration present on the tooth ^33^. Composite resin will be used as restorative material for the new restoration.

The presence or absence of soft or hard carious tissue is evaluated and recorded by the postgraduate dental student who provides dental care after the restoration removal when replacement is indicated. Training and calibration were conducted before the assessments. An experienced researcher in Cariology performed a theoretical lecture about the clinical characteristics of caries lesions, and training was carried out using photos of clinical cases. The procedure of evaluating the carious tissue is performed to record a possible false-positive diagnosis for dentine caries lesion around the restoration. The authors will also develop an accuracy study nested in this clinical trial.

**Figure 4.**
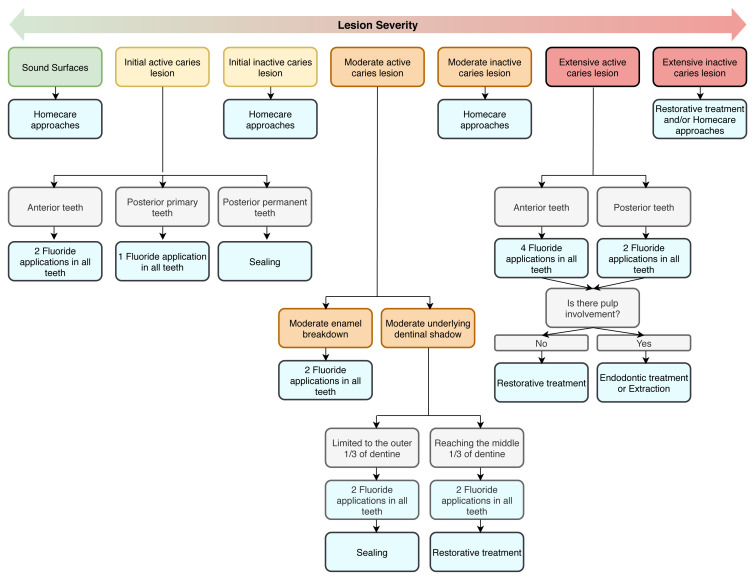
Patient’s plan decision flowchart based on the International Caries Detection and Assessment System (ICDAS) criteria.

The same operators are performing additional dental treatment needs (not related to the restorations included in the study). Treatment plan related to additional dental treatment was carried out by the examiner responsible for children's initial clinical examination. Details of the pre-established treatment protocols can be found in Figure 4.

### Follow-up visits

After the completion of the treatment plan, participants will be followed up considering the outcome evaluation after six, 12, 18, and 24 months. At the follow-up visits, if a new dental treatment is needed (related or not to the restorations), necessary procedures will be carried out. Hygiene and dietary instructions will be given to children at each follow-up visit.

The treatment decisions for the restorations evaluated during the follow-up visits will be decided according to the FDI or CARS criteria, considering the child’s allocation group. The same trained and calibrated examiner (BLPM) who conducted the assessments at the beginning of the study, with the FDI or CARS criteria, will perform the assessments with the FDI or CARS criteria during all follow-up visits.

During the 24 months follow-up visit, a new ECOHIS questionnaire will be applied for parents or legal guardians who had previously answered at the time the child was included in the study.

### Adherence

Stimuli for participants' adherence to the treatment and follow-up sessions are happening via mobile and social networks. Facebook and Instagram profiles were created to stay in touch with patients through social media. Humanized care is provided for all participants, focusing on the patient's well-being and providing empathy, affection, and familiarity between the CARDEC collaborative group and children and their families. Explanations about the importance of participation for their benefit are also being given.

### Outcomes

The primary outcome of this trial will be the need for a new intervention during the follow-up of restorations evaluated by different criteria. This outcome consists of several components. Thus, the outcome occurrence will be considered if any of the following conditions are detected:

- Presence of secondary caries lesion exposing dentin;
- Need for repair;
- Need for restoration replacement;
- Need for extension of the existing restoration on the examined tooth due to a tooth fracture or caries lesion development exposing dentin;
- An episode of pain or need for endodontic treatment;
- Extraction requirement (except in the case of prolonged retention).

The occurrence of any of these conditions at any time of follow-up will be considered as an event related to the primary outcome. Each of the events that make up the primary outcome will be analyzed separately as secondary outcomes. Changes in children's oral health-related quality of life after two years will be considered as a secondary outcome. The costs and effects per child of the treatments performed during the follow-up, considering the teeth included in our sample, are also going to be analyzed as a secondary outcome.

The occurrence of the outcomes will be evaluated according to predetermined criteria from two other criteria during the follow-up visits of six, 12, 18, and 24 months. Different criteria will be used according to the number of surfaces the restoration involves:

- For one-surface restorations: the criteria used will be according to Frencken *et al*. ^34^;
- For a multi-surface restoration: the criteria used will be according to Roeleveld *et al*. ^35^.

According to Frencken *et al*. ^34^ criteria, scores related to restoration success will be 0, 1 or 7. Those considered to have failed will be scored as 2, 3, 4 or 8; while those considered being unrelated to success and failure will be scored as 5, 6 or 9. Concerning the Roeleveld *et al*. ^35^ criteria, restoration success will be scored as 00 or 10. Those considered to have failed will be scored as 11, 12, 13, 20, 21, 30 or 40; while those considered being unrelated to success and failure will be scored as 50, 60, 70 or 90.

The information regarding presence of secondary caries lesions exposing dentin; the need for repair; the need for restoration replacement; the need for extension of the existing restoration on the examined tooth; the need for endodontic treatment, and extraction requirement are obtained directly using the criteria systems proposed (Frencken *et al*., and Roeleveld *et al*.). In cases of suspected pulp involvement, a radiograph is taken. We also asked the parents about pain occurrence.

The follow-up evaluations will be carried out by an examiner (TKT) blind to the children’s allocation group. The examiner was previously trained and calibrated for both criteria (the weighted Kappa value for interexaminer was 0.89, and the intra-examiner agreement was 0.94). The researcher (TKT) did not participate in the previous phases of the trial and will perform the evaluations according to the Frencken et al. or Roeleveld et al. during all follow-up visits (six, 12, 18, and 24 months), considering the number of restorations surface, to assess the outcome of the study.

### Sample size

The sample size calculation was performed based on the primary outcome (percentage of restorations requiring reintervention). A failure rate of 10% after two years was considered for occlusal restorations ^36^ and 30% for occlusal-proximal restorations ^37^. It was also considered that approximately 10% of the replaced restorations and 14% of the restorations undergoing repair fail again ^38^. Considering that half of the sample is occlusal restorations, an operative reintervention requirement rate of 24% is expected in two years. The minimum number of 522 restorations was reached, based on an absolute difference of 10% between the groups, using a two-tailed test. As a child can contribute with more than one restoration, 20% was added to the sample size (n = 626).

Considering that children with restored teeth have on average 3.7 restorations ^39^, and adding 20% for possible participants loss, a minimum number of 204 children presenting at least one restored primary tooth (without fistula, abscess, pulp exposure, history of spontaneous dental pain or mobility) is required to be included in this trial.

## Data management and analysis

### Data management

Clinical data will be entered directly into predetermined sheets. Data quality will be ensured by validation checks that include missing data, out-of-range values, and illogical and invalid responses.

### Statistical methods

Examiners' reproducibility will be performed using the weighted kappa test, calculating the weighted value of kappa and also the 95% confidence intervals. The primary outcome of the study is a dichotomous variable (with or without the need for intervention); therefore, the unit of analysis is the restored tooth. As children can have more than one tooth included in the study, the comparison between the groups will be carried out using survival analysis, considering the cluster-effect. Kaplan-Meyer graphs will be constructed, and the methods will be compared using the Cox regression model with a shared frailty.

Secondary clinical outcomes will also be analyzed using the same statistical tests. Quality of life will be analyzed using Poisson regression analysis and the unit of analysis will be the child.

A trial-based economic evaluation will be performed considering the difference of the inputs (costs) and outputs (effects) of the two diagnostic criteria (FDI and CARS) after two years. Further details regarding the economic evaluation will be described on a health economic analysis plan.

A p-value of 5% as the level of significance will be considered for all tests. The analyses will be performed using the statistical package Stata 13.0 (Stata Corp, College Station, USA).

## Participant recruitment and timeline

**Figure 5.**
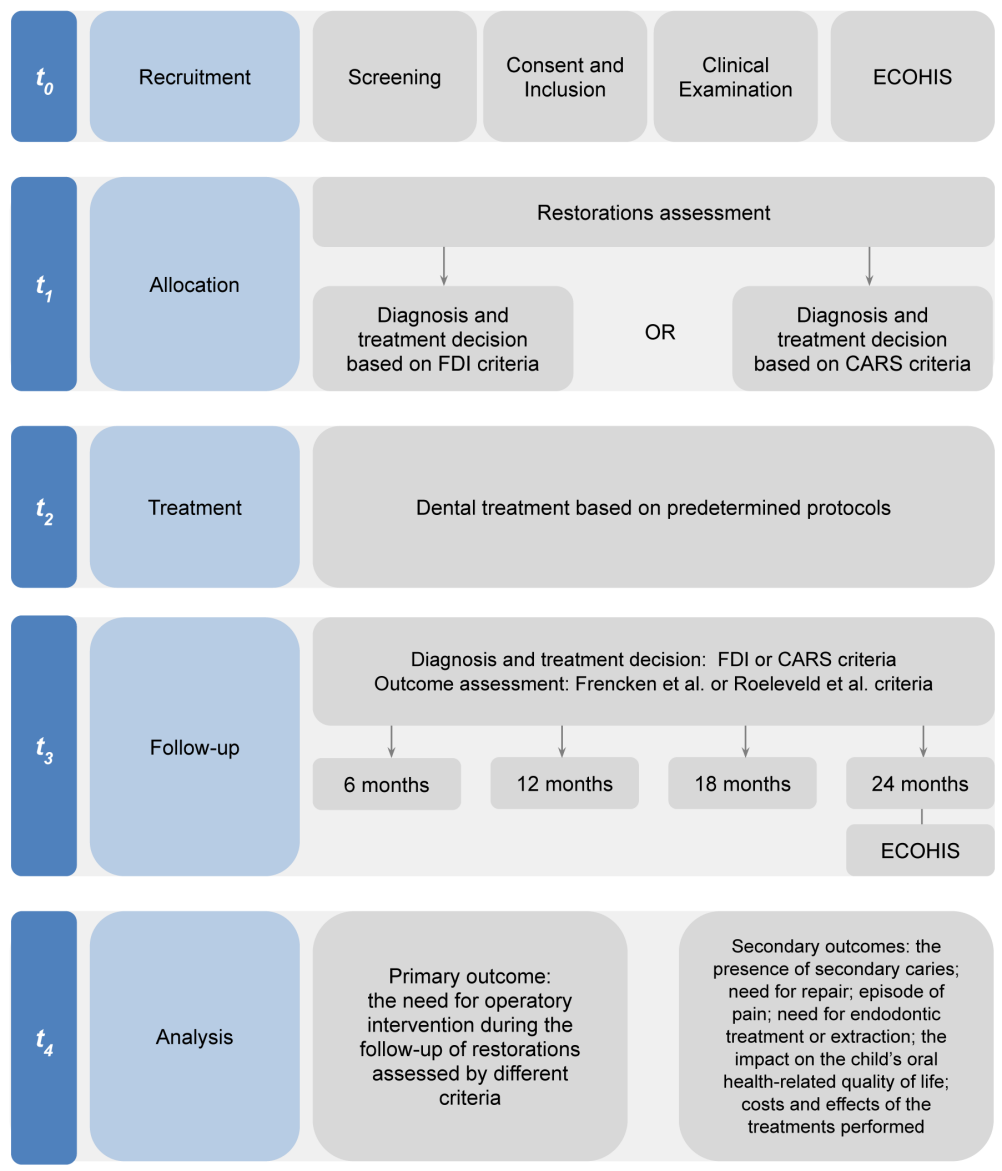
Clinical trial's timeline. ECOHIS, Early Childhood Oral Health Impact Scale; FDI, International Dental Federation; CARS, Caries Associated with Restorations and Sealants.

Recruitment took place at the School of Dentistry of the University of São Paulo from November 2017 to November 2018. Each allocated participant will have an average treatment period of one month and will be followed-up for 24 months, resulting in a total of 25 months of enrollment. The detailed timeline for data collection is summarized in Figure 5.

## Monitoring

### Data monitoring

No data monitoring committee is needed in this trial since adverse events are unlikely to happen during restoration evaluation and dental treatments. For this reason, the chief investigator of the study (FMM) will assume an independent oversight of trial data collection, management, and analysis. 

### Harms

The effects expected in this study are the ones listed as trial outcomes. All of them are usually expected to happen during pediatric dentistry clinical practice. Any other adverse event is unlikely to happen.

### Auditing

The data will be periodically subjected to audit by the coordinator of the study. Any discrepancies will be verified, corrected and registered.

## Ethics and dissemination

### Confidentiality

Sequential numbers will be used to identify and ensure participant confidentiality. Participants’ identifiable information will be stored in filing cabinets in a locked secure room.

### Access to data

The full data generated from this trial will be placed in a public repository ( University of São Paulo Data Repository).

### Ancillary and post-trial care

Participants included in this trial will have dental treatments provided at the School’s dental clinic during and after the completion of the trial if necessary.

### Dissemination policy

All the findings of this trial will be reported in peer-reviewed journals, patient newsletters and the School of Dentistry of University of São Paulo website.

## Study status

The patient recruitment took place from 16 November 2017 to 30 November 2018. The follow-up evaluations of 6 and 12 months were concluded; however, the study is now temporarily suspended since 16 March 2020 due to COVID-19. 

## Discussion

Restoration assessment is a challenge in dentistry, and the main point of debate is caries around restoration ^1, 40^. However, due to the scarcity of well-conducted studies, its diagnosis is not based on objective clinical criteria, and there is a considerable variation in the criteria used. As a consequence, a significant number of restorations presenting small defects are often indicated to be replaced since they can be misdiagnosed as caries lesions ^9^. Also, there is no homogeneity on the treatment decision-making for secondary caries between dentists ^5, 41^, and studies based on clinical practice have shown that they tend to replace more restorations than necessary ^42^.

Two recently published systematic reviews included around 20 accuracy studies of methods for detecting caries lesions around restorations ^5, 13^. The majority of these studies were performed *in vitro*, assessed caries lesions in permanent teeth, and did not evaluate relevant aspects to the clinical practice ^5, 13^. Nevertheless, the decision on what is the best method to be used should evaluate whether patients undergoing such methods would have greater health-related benefits than patients undergoing some other method ^43^. For this assessment, ultimate health outcomes for patients must be considered. The experimental design to assess it is the randomized clinical trial (Phase IV question).

Randomized clinical trials are considered the best study design on which clinicians and policy-makers rely most to determine whether an intervention is effective ^44^. However, as far as we know, no randomized clinical study has been carried out regarding the detection of caries lesions around restorations in primary teeth. Besides that, no study compared the accuracy of FDI and CARS criteria clinically to detect caries around restoration on primary teeth, and the impact of the use of the criteria on the restorative treatment decisions for children. For this reason, an accuracy study (Phase III question) with the FDI and CARS methods will be developed nested to this trial.

For the present trial, the authors decided to use among the FDI criteria the subcategories marginal staining and marginal adaptation, beyond recurrence of caries. The decision was based on the fact that both aspects can be misinterpreted with secondary caries during restoration assessment ^45– 47^. Therefore, we tried to simulate what can clinically be a reason for restoration reintervention in the daily clinical practice. Regarding the CARS criteria, the system does not present any treatment decision linked to the evaluation method. For this reason, we adapted the decisions based on the ICCMS recommendations for treating primary caries lesions ^48^.

The criteria systems used to assess the study outcome, although different, were defined mainly to evaluate our primary endpoint, which is the necessity of replacement of the restoration. The difference between the two criteria is because one is used to assessing one-surface restorations (Frencken *et al*.), and the other is used for assessing multi-surface restoration (Roeleveld *et al*.). However, both are used to evaluate the necessity of restoration replacement. Regarding the patient perspective, the reason that led to the replacement probably is not important. We could assess this information with some patient-reported variables (or proxies, reported by the parents). The suitability of the two criteria for the dentists will not be evaluated in our study. Still, we can speculate about this topic in the main manuscript after obtaining the results.

The study’s limitation is that the first assessment performed with the participant’s allocation group (FDI or CARS criteria) and the second assessment according to the other criteria will be done at the same dental appointment. This will be done to reduce the number of dental appointments for the patients, enhancing their adherence to the clinical research. However, a carry-over effect could occur between the methods. Contrariwise, a strength of the study is the procedure used to avoid selection bias. The evaluations will be conducted in a sample of children randomly selected from a list of patients who sought dental treatment at our School. Besides that, the outcome assessor will be blinded regarding the allocation group to avoid assessment bias.

Thus, with the development of this clinical trial and expected results, we aim to define between FDI and CARS criteria the best approach for diagnosis and management of dental restorations in children, considering the impact on the treatment decision on clinically relevant outcomes for the patient and costs resulting from the treatments performed.

## Data availability

### Underlying data

No underlying data are associated with this article.

### Extended data

Figshare: Consent form. https://doi.org/10.6084/m9.figshare.12327644.v1 ^16^.

Figshare: Consent form in the original language (Portuguese). https://doi.org/10.6084/m9.figshare.12327674.v1 ^18^.

Figshare: Assent form. https://doi.org/10.6084/m9.figshare.12327731.v1 ^17^.

Figshare: Assent form in the original language (Portuguese). https://doi.org/10.6084/m9.figshare.12327779.v1 ^19^.

Figshare: Restorations assessment form. https://doi.org/10.6084/m9.figshare.12331460.v1 ^22^.

Figshare: Restorations assessment form in the original language (Portuguese).

https://doi.org/10.6084/m9.figshare.12331466.v1 ^23^.

Figshare: Anamnesis form. https://doi.org/10.6084/m9.figshare.12324212.v1 ^27^.

Figshare: Anamnesis form in the original language (Portuguese). https://doi.org/10.6084/m9.figshare.12327578.v1 ^28^.

Figshare: Time and cost form. https://doi.org/10.6084/m9.figshare.12327854.v1 ^31^.

Figshare: Time and cost form in the original language (Portuguese). https://doi.org/10.6084/m9.figshare.12331451.v1 ^32^.

### Reporting guidelines

Figshare: SPIRIT checklist. https://doi.org/10.6084/m9.figshare.12331484.v1 ^15^.

Data are available under the terms of the Creative Commons Zero “No rights reserved” data waiver (CC0 1.0 Public domain dedication).
