# Supplementary material for: The effect of two clinical criteria in the assessment of caries lesions around restorations in children (CARDEC-03): study protocol for a diagnostic randomized clinical trial
Source: F1000Res. 2021 Jan 11;9:650. Originally published 2020 Jun 26. [Version 3] doi: 10.12688/f1000research.23801.3 (PMC7818095; doi:10.12688/f1000research.23801.3)
Supplement: Peer review history

# Peer review: Reviewer response for version 3

**DOI:** 10.5256/f1000research.34232.r77010

**Published:** 2021-01-20

**Authors:** Andrea Zandoná

**Affiliations:** 1 Department of Comprehensive Care, Tufts University School of Dental Medicine, Boston, MA, USA

Thank you for your responses. The authors have addressed all comments.

Is the study design appropriate for the research question?

Yes

Is the rationale for, and objectives of, the study clearly described?

Yes

Are sufficient details of the methods provided to allow replication by others?

Partly

Are the datasets clearly presented in a useable and accessible format?

Not applicable

Reviewer Expertise:

Cariology

I confirm that I have read this submission and believe that I have an appropriate level of expertise to confirm that it is of an acceptable scientific standard.

# Response

**Published:** 2021-01-20

**Authors:** Fausto Mendes

**Affiliations:** School of Dentistry of the University of São Paulo, Brazil

Dear prof. Zandoná

 I would like to thanks for your excellent suggestions. We are very grateful for your contribution.

 Best regards

 Fausto

# Peer review: Reviewer response for version 3

**DOI:** 10.5256/f1000research.34232.r77009

**Published:** 2021-01-11

**Authors:** Ana Paula Pires dos Santos

**Affiliations:** 1 School of Dentistry, Department of Community and Preventive Dentistry, University of the State of Rio de Janeiro - UERJ, Rio de Janeiro, Brazil

All the points raised have been addressed satisfactorily.

Is the study design appropriate for the research question?

Yes

Is the rationale for, and objectives of, the study clearly described?

Yes

Are sufficient details of the methods provided to allow replication by others?

Partly

Are the datasets clearly presented in a useable and accessible format?

Not applicable

Reviewer Expertise:

Cariology and Epidemiology

I confirm that I have read this submission and believe that I have an appropriate level of expertise to confirm that it is of an acceptable scientific standard.

# Response

**Published:** 2021-01-12

**Authors:** Fausto Mendes

**Affiliations:** School of Dentistry of the University of São Paulo, Brazil

Dear Prof. Ana Paula

 We are very grateful for your valuable suggestions and comments provided. They improved significantly our text. Thank you very much.

 Best Wishes

 The authors

# Peer review: Reviewer response for version 2

**DOI:** 10.5256/f1000research.29958.r71876

**Published:** 2020-11-26

**Authors:** Ana Paula Pires dos Santos

**Affiliations:** 1 School of Dentistry, Department of Community and Preventive Dentistry, University of the State of Rio de Janeiro - UERJ, Rio de Janeiro, Brazil

This is an interesting and relevant study. I congratulate the authors for the initiative and acknowledge all the efforts needed for conducting this study. However, I have some suggestions that I believe may help readers better understand the rationale and methods employed.

**Introduction**

 The authors pointed out that the FDI criteria evaluates some aspects that might not be directly related to caries lesions, such as “marginal staining” and “marginal adaptation”. If the aim was to compare two different criteria for diagnosis of caries around restorations, why not focus only on the “recurrence of caries” aspect?

**Study setting**

 The authors stated that the participants were randomly selected from a list of patients who sought dental treatment at the School of Dentistry. Were participants randomly selected or were participants that fulfilled the eligibility criteria randomly allocated to the intervention groups?

**Participant eligibility**

 I would suggest that inclusion criterion **c** should read as follows: Presenting at least one restoration of any restorative material (composite resin, amalgam or glass ionomer cement), regardless of its condition, on a primary tooth (anterior or posterior) without fistula, abscess, pulp exposure, history of spontaneous dental pain or mobility.

**Allocation: sequence generation and concealment mechanism**

 I would suggest authors should rewrite the randomization mechanism. When a stratified randomization using blocks is used, firstly participants are stratified in different strata; then randomization using blocks (in this case, of different sizes) is performed within each stratum. So, the sentence “Randomization was stratified by blocks…” may not accurately describe what happened. Also, authors should clarify whether random permuted block sizes were used.

**Interventions**

 The authors said both criteria were used in all children in order to compare the methods of the study. But as this is an RCT, wouldn’t it be possible to compare the methods even if children received only the diagnosis criterion they were allocated to?

 The ECOHIS is intended to assess the impact on preschool children’s oral health-related quality of life, but this study included children from three to 10 years-old. Please clarify this issue.

**Follow-up visits and outcomes**

1. The authors stated that participants will be followed up considering the outcome evaluation after six, 12, 18, and 24 months and that the treatment decisions for the restorations evaluated during the follow-up visits will be decided according to the FDI or CARS criteria, considering the child’s allocation group.
2. They also stated that the occurrence of the outcomes will be evaluated according to predetermined criteria from two other criteria during the follow-up visits of six, 12, 18, and 24 months. Different criteria will be used according to the number of surfaces the restoration involves: for one-surface restorations: the criteria used will be according to Frencken *et al*. and for a multi-surface restoration: the criteria used will be according to Roeleveld *et al.*

Please clarify the differences between these evaluations. Is the first related to teeth not included in the trial and the latter to teeth included in the trial?

 Also, it is written that the same trained and calibrated examiner (BLPM) who conducted the assessments at the beginning of the study will perform the evaluations. It is also written that the follow-up evaluations will be carried out by an examiner (TKT) blind to children’s allocation group who was previously trained and calibrated for both criteria and not participating in the previous phases of the trial. Please clarify again the differences between these evaluations.

 Lastly, it is not clear how the outcome is measured: when the following conditions are detected (presence of secondary caries lesion exposing dentin; need for repair; need for restoration replacement; need for extension of the existing restoration on the examined tooth due to a tooth fracture or caries lesion development exposing dentin; an episode of pain or need for endodontic treatment; extraction requirement (except in the case of prolonged retention) OR according to the criteria proposed by Frencken *et al*. and Roeleveld *et al*.?

 I believe it is important to mention the calibration scores for the outcome assessment.

**Sample size**

 Please consider adding the sentence in italic:

 Considering that children with restored teeth have on average 3.7 restorations ^38^, and adding 20% for possible participants loss, a minimum number of 204 children presenting at least one restored primary tooth - *without fistula, abscess, pulp exposure, history of spontaneous dental pain or mobility - *is required to be included in this trial.

**Discussion**

 The interventions consisted of two diagnosis criteria for “caries around restorations” whereas outcome was assessed using two criteria for “restoration success”. What are the differences between these two sets of criteria? Aren’t them all essentially aiming to assess whether a restoration should be replaced? From the patient’s perspective, is the reason that led to the replacement of a restoration important? From the dentist’s perspective, which criterion is more suitable: one that assess caries around restorations or one that access the success of restorations? Please consider discussing these aspects.

**Table 3 **- minor correction

 Please insert “in” in the sentence “Replacement should be indicated in case the carious lesion involves more than half of the restoration”.

**Figure 2 **- minor correction

 t0: screening not screeing.

Is the study design appropriate for the research question?

Yes

Is the rationale for, and objectives of, the study clearly described?

Yes

Are sufficient details of the methods provided to allow replication by others?

Partly

Are the datasets clearly presented in a useable and accessible format?

Not applicable

Reviewer Expertise:

Cariology and Epidemiology

I confirm that I have read this submission and believe that I have an appropriate level of expertise to confirm that it is of an acceptable scientific standard, however I have significant reservations, as outlined above.

# Response

**Published:** 2020-12-22

**Authors:** Fausto Mendes

**Affiliations:** School of Dentistry of the University of São Paulo, Brazil

Dear Professor Ana Paula Pires dos Santos. We are grateful for the comments and suggestions provided. To facilitate the review process, your comments are summarized before the respective answers.

**Reviewer: Introduction**

**The authors pointed out that the FDI criteria evaluates some aspects that might not be directly related to caries lesions, such as “marginal staining” and “marginal adaptation”. If the aim was to compare two different criteria for diagnosis of caries around restorations, why not focus only on the “recurrence of caries” aspect?**

 Our response: Dear Professor Ana Paula Pires dos Santos, the following phrase was added to the manuscript to clarify this point (Introduction, page 3, 2nd paragraph): “However, these aspects could be relevant to be evaluated when using the FDI criteria since many dentists and studies associate marginal staining and defects in the marginal adaptation with the presence of caries lesion around the restoration”. A new reference was also added to this paragraph: Signori C, Gimenez T, Mendes FM, Huysmans M-CDNJM, Opdam NJM, Cenci MS. Clinical relevance of studies on the visual and radiographic methods for detecting secondary caries lesions - a systematic review. J Dent.2018. https://doi.org/10.1016/j.jdent.2018.05.018.

**Reviewer: Study setting**

**The authors stated that the participants were randomly selected from a list of patients who sought dental treatment at the School of Dentistry. Were participants randomly selected or were participants that fulfilled the eligibility criteria randomly allocated to the intervention groups?**

 Our response: Dear reviewer, thank you for your suggestion. The necessary changes were made to the manuscript (Methods, page 5, 3rd paragraph): “The participants (three to 10 years old) were selected from a list of patients who sought dental treatment at the School of Dentistry. Those that fulfilled the eligibility criteria were randomly allocated to the intervention groups”.

**Reviewer: Participant eligibility**

**I would suggest that inclusion criterion c should read as follows: Presenting at least one restoration of any restorative material (composite resin, amalgam or glass ionomer cement), regardless of its condition, on a primary tooth (anterior or posterior) without fistula, abscess, pulp exposure, history of spontaneous dental pain or mobility.**

 Our response: Dear reviewer, the inclusion criterion was written as you suggested (Methods, page 6, 2nd paragraph): “Presenting at least one restoration of any restorative material (composite resin, amalgam or glass ionomer cement), regardless of its condition, on a primary tooth (anterior or posterior) without fistula, abscess, pulp exposure, history of spontaneous dental pain or mobility”.

**Reviewer: Allocation: sequence generation and concealment mechanism**

**I would suggest authors should rewrite the randomization mechanism. When a stratified randomization using blocks is used, firstly participants are stratified in different strata; then randomization using blocks (in this case, of different sizes) is performed within each stratum. So, the sentence “Randomization was stratified by blocks…” may not accurately describe what happened. Also, authors should clarify whether random permuted block sizes were used.**

 Our response: Dear reviewer, the randomization mechanism was rewritten as you suggested (Methods, page 7, 1st paragraph): “Firstly, participants were stratified into different strata: (1) children aged 3 to 6 years presenting three restorations or less; (2) children aged 7 to 10 years presenting three restorations or less; (3) children aged 3 to 6 years presenting more than three restorations; (4) children aged 7 to 10 years presenting more than three restorations. The number of restorations considered for stratification was those placed in primary and permanent teeth. Then, randomization using blocks of different sizes (2, 4, 6 or 8) was performed within each stratum”.

**Reviewer: Interventions**

**The authors said both criteria were used in all children in order to compare the methods of the study. But as this is an RCT, wouldn’t it be possible to compare the methods even if children received only the diagnosis criterion they were allocated to?**

 Our response: Dear reviewer, the following sentence was added to the manuscript to clarify this point (Interventions, page 9, 5th paragraph): “This procedure aims to compare the methods of the study since a cross-sectional study was developed nested in this randomized clinical trial”. The diagnostic methods have been already compared and the results were published this year: Moro BLP, Freitas RD, Pontes LRA, Pássaro AL, Lenzi TL, Tedesco TK, Ekstrand KR, Braga MM, Raggio DP, Cenci MS, Mendes FM. Influence of different clinical criteria on the decision to replace restorations in primary teeth. J Dent. 2020 Oct;101:103421. doi: 10.1016/j.jdent.2020.103421. Epub 2020 Jun 29. PMID: 32615237.

**Reviewer: The ECOHIS is intended to assess the impact on preschool children’s oral health-related quality of life, but this study included children from three to 10 years-old. Please clarify this issue.**

 Our response: Dear reviewer, we decided to use an instrument answered by the parents since our sample's age range is large and involves small children who would have difficulty answering other questionnaires (CPQ8-10, for example). This choice was made to allow data to be collected for the entire sample and for the same instrument to be standardized. We have already used ECOHIS for older children in previous longitudinal studies (Guedes et al., Community Dent Oral Epidemiol 2016; 44:292; Guedes et al., Int J Paediatr Dent 2018; 28:207). This explanation was added to the manuscript (Interventions, page 10, 2nd paragraph).

**Reviewer: Follow-up visits and outcomes**

1. **The authors stated that participants will be followed up considering the outcome evaluation after six, 12, 18, and 24 months and that the treatment decisions for the restorations evaluated during the follow-up visits will be decided according to the FDI or CARS criteria, considering the child’s allocation group.**
2. **They also stated that the occurrence of the outcomes will be evaluated according to predetermined criteria from two other criteria during the follow-up visits of six, 12, 18, and 24 months. Different criteria will be used according to the number of surfaces the restoration involves: for one-surface restorations: the criteria used will be according to Frencken et al. and for a multi-surface restoration: the criteria used will be according to Roeleveld et al.**

**Please clarify the differences between these evaluations. Is the first related to teeth not included in the trial and the latter to teeth included in the trial?**

 Our response: Dear reviewer, we are just talking about teeth included in the trial. However, as it is a diagnostic study, we decided to use different methods (and not the FDI and CARS again) to evaluate the study's outcome. For this reason, the Frencken et al. and the Roeleveld et al. are being used in the evaluations performed in the follow-up appointments.  Nevertheless, we also repeated the assessment using the allocation group during all follow-up visits because "when it comes to the management of restorations in primary dentition, it is impossible to know if a more conservative or invasive approach would bring more benefits to children". We believe that the FDI "may lead to a more interventional approach" and the CARS criteria "is probably more conservative when it comes to restoration reintervention". So, as a longitudinal study is being carried out, if we repeated the assessments and made the treatment decisions with each diagnostic method, we could also evaluate if a more conservative or invasive approach would benefit children, according to the treatment decisions of the FDI and CARS criteria. We did not add this explanation in the manuscript, as we believe that with the other changes, this issue was also clarified.

**Reviewer: Also, it is written that the same trained and calibrated examiner (BLPM) who conducted the assessments at the beginning of the study will perform the evaluations. It is also written that the follow-up evaluations will be carried out by an examiner (TKT) blind to children’s allocation group who was previously trained and calibrated for both criteria and not participating in the previous phases of the trial. Please clarify again the differences between these evaluations.**

 Our response: Dear reviewer, new information was provided to clarify the differences between the evaluations:

1. The treatment decisions for the restorations evaluated during the follow-up visits will be decided according to the FDI or CARS criteria, considering the child’s allocation group. The same trained and calibrated examiner (BLPM) who conducted the assessments at the beginning of the study, with the FDI or CARS criteria, will perform the assessments with the FDI or CARS criteria during all follow-up visits.
2. The follow-up evaluations will be carried out by an examiner (TKT) blind to the children’s allocation group. The examiner was previously trained and calibrated for both criteria (the weighted Kappa value for interexaminer was 0.89, and the intra-examiner agreement was 0.94). The researcher (TKT) did not participate in the previous phases of the trial and will perform the evaluations according to the Frencken et al. or Roeleveld et al. during all follow-up visits (six, 12, 18, and 24 months), considering the number of restorations surface, to assess the outcome of the study.

**Reviewer: Lastly, it is not clear how the outcome is measured: when the following conditions are detected (presence of secondary caries lesion exposing dentin; need for repair; need for restoration replacement; need for extension of the existing restoration on the examined tooth due to a tooth fracture or caries lesion development exposing dentin; an episode of pain or need for endodontic treatment; extraction requirement (except in the case of prolonged retention) OR according to the criteria proposed by Frencken et al. and Roeleveld et al.?**

 Our response: The information regarding the presence of secondary caries lesions exposing dentin; the need for repair; the need for restoration replacement; the need for extension of the existing restoration on the examined tooth; the need for endodontic treatment, and extraction requirement are obtained directly using the criteria systems proposed (Frencken et al., and Roeleveld et al.). In cases of suspected pulp involvement, a radiograph is taken. We also asked the parents about pain occurrence. We added this information in the text (Outcomes, page 13, 6th paragraph).

**Reviewer: I believe it is important to mention the calibration scores for the outcome assessment.**

 Our response: Dear reviewer, the information was provided (Outcomes, page 14, 3rd paragraph): “the weighted Kappa value for interexaminer was 0.89, and the intra-examiner agreement was 0.94”.

**Reviewer: Sample size**

**Please consider adding the sentence in italic:**

**Considering that children with restored teeth have on average 3.7 restorations [38], and adding 20% for possible participants loss, a minimum number of 204 children presenting at least one restored primary tooth - *without fistula, abscess, pulp exposure, history of spontaneous dental pain or mobility* - is required to be included in this trial.**

 Our response: Dear reviewer, the sentence was added to the manuscript (Sample size, page 15, 2nd paragraph): “Considering that children with restored teeth have on average 3.7 restorations [38], and adding 20% for possible participants loss, a minimum number of 204 children presenting at least one restored primary tooth (without fistula, abscess, pulp exposure, history of spontaneous dental pain or mobility) is required to be included in this trial”.

**Reviewer: Discussion**

**The interventions consisted of two diagnosis criteria for “caries around restorations” whereas outcome was assessed using two criteria for “restoration success”. What are the differences between these two sets of criteria? Aren’t them all essentially aiming to assess whether a restoration should be replaced? From the patient’s perspective, is the reason that led to the replacement of a restoration important? From the dentist’s perspective, which criterion is more suitable: one that assess caries around restorations or one that access the success of restorations?** **Please consider discussing these aspects.**

 Our response: Dear reviewer, we add a paragraph to discuss these aspects in the manuscript (Discussion, page 20, 3rd paragraph):  The criteria systems used to assess the study outcome, although different, were defined mainly to evaluate our primary endpoint, which is the necessity of replacement of the restoration. The difference between the two criteria is because one is used to assessing one-surface restorations (Frencken et al.), and the other is used for assessing multi-surface restoration (Roeleveld et al.). However, both are used to evaluate the necessity of restoration replacement. Regarding the patient perspective, the reason that led to the replacement probably is not important. We could assess this information with some patient-reported variables (or proxies, reported by the parents). The suitability of the two criteria for the dentists will not be evaluated in our study. Still, we can speculate about this topic in the main manuscript after obtaining the results.

**Reviewer: Table 3 - minor correction**

**Please insert “in” in the sentence “Replacement should be indicated in case the carious lesion involves more than half of the restoration”.**

 Our response: Dear reviewer, the word was added to the sentence.

**Reviewer: Figure 2 - minor correction**

**t0: screening not screeing.**

 Our response: Dear reviewer, thank you for your suggestion. The spelling was corrected.

# Peer review: Reviewer response for version 1

**DOI:** 10.5256/f1000research.26263.r65636

**Published:** 2020-07-20

**Authors:** Andrea Zandoná

**Affiliations:** 1 Department of Comprehensive Care, Tufts University School of Dental Medicine, Boston, MA, USA

The aim of this study is to evaluate the effect of two different visual criteria, the FDI and CARS criteria for the assessment of caries around restorations in primary teeth on outcomes related to children’s oral health and costs.

 This is a timely study that is assessing an important topic from the outcomes point of view, which makes it very clinically relevant. The study protocol is well written, provides a rationale, and clearly describes the objectives of the study. The study design is appropriate for the research question. There are however insufficient details of the methods to allow replication by others.

 The following should be clarified:

 Given the impact that caries risk can have on the outcomes, it is important to understand if caries risk was assessed and played any role in randomization. Additionally, although caries lesion activity was assessed, it is not clear if randomization considered caries lesion activity status. It is also not clear if exfoliation status was considered and how that affected inclusion/exclusion. Regarding the 4 stratification blocks, please clarify if the number of restorations considered were only those in the primary dentition or if the restorations in the permanent dentition were also considered for stratification.

 On examiner calibration – please clarify if the repeat examinations included all 10 children. It is stated that examinations of study patients only occurred after weighted values were greater than 0.75. Please clarify if the same 10 children were repeatedly examined to achieve these values for both intra- and inter-examiner reliability. Were new Kappa scores calculated for new exams or were the exams compounded and the Kappa scores re-calculated? After removal of the restoration when the replacement was indicated the hardness of dentin was evaluated, please clarify if this was done by the provider or by a different examiner and if the calibration was conducted prior to the assessments.

 On Adherence, the authors state that “humanized care is provided for all participants. Please provide a definition of “humanized care”.

 Management:

 Figure 1 provides a very clear flow chart of how lesions were managed according to the ICDAS criteria. A similar chart with the FDI criteria and management would be helpful. Additionally, the predetermined protocols need to be overlaid with the FDI and CARS to better illustrate how the different cases were/are handled.

Is the study design appropriate for the research question?

Yes

Is the rationale for, and objectives of, the study clearly described?

Yes

Are sufficient details of the methods provided to allow replication by others?

Partly

Are the datasets clearly presented in a useable and accessible format?

Not applicable

Reviewer Expertise:

Cariology

I confirm that I have read this submission and believe that I have an appropriate level of expertise to confirm that it is of an acceptable scientific standard, however I have significant reservations, as outlined above.

# Response

**Published:** 2020-09-28

**Authors:** Fausto Mendes

**Affiliations:** School of Dentistry of the University of São Paulo, Brazil

Dear Professor Andrea Zandoná. We are grateful for the comments and suggestions provided. In order to facilitate the review process, your comments are summarized before the respective answers.

**Reviewer: Given the impact that caries risk can have on the outcomes, it is important to understand if caries risk was assessed and played any role in randomization. Additionally, although caries lesion activity was assessed, it is not clear if randomization considered caries lesion activity status. It is also not clear if exfoliation status was considered and how that affected inclusion/exclusion.**

 Our response: Dear Professor Andrea Zandoná, the following paragraph was added to the manuscript to clarify these points (Methods, page 7, 1st paragraph): “All participants of the study could be classified as having a high caries risk since past caries experience is the most important component for the development of caries lesions. However, stratified randomization was performed considering the children's number of restorations to subdivide them in children with higher and lower caries experience. The caries lesion activity was not considered for randomization.  On the other hand, the children's age was a parameter for stratification in order to consider the different time of exfoliation of the evaluated teeth. In this way, the number of teeth with different times of exfoliation was balanced between the FDI and CARS criteria”. A new reference was also added to this paragraph: Twetman S, Fontana M, Featherstone JD. Risk assessment - can we achieve consensus?. Community Dent Oral Epidemiol. 2013;41(1):e64-e70. doi:10.1111/cdoe.12026

 Regarding considering the exfoliation status in inclusion and exclusion criteria, we excluded from the sample restorations on teeth with mobility. This is how the exfoliation status affected the exclusion criteria of the study (Methods, page 6, 3rd paragraph).

**Reviewer: Regarding the 4 stratification blocks, please clarify if the number of restorations considered were only those in the primary dentition or if the restorations in the permanent dentition were also considered for stratification.**

 Our response: Dear reviewer, we added a sentence to explain that “the number of restorations considered for stratification were those placed in primary and permanent teeth” (Methods, page 6, 4th paragraph).

**Reviewer: On examiner calibration – please clarify if the repeat examinations included all 10 children. It is stated that examinations of study patients only occurred after weighted values were greater than 0.75. Please clarify if the same 10 children were repeatedly examined to achieve these values for both intra- and inter-examiner reliability. Were new Kappa scores calculated for new exams or were the exams compounded and the Kappa scores re-calculated?**

 Our response: Dear reviewer, thank you for your suggestions. Two sentences were added to answer your question: “After these procedures, the examiner evaluated restorations in 10 children who did not participate in the clinical trial. The examiner repeated the same evaluation, in the same 10 children, for intra-examiner agreement. A benchmark examiner (TLL) also performed the tests to assess inter-examiner reproducibility twice in the same sample of children. In this way, the exams were compounded, and the weighted kappa scores were re-calculated. The assessment of children included in the study started after the intra-examiner and inter-examiner weighted kappa value reached values greater than 0.75 for both FDI and CARS criteria” (Methods, pages 8 and 9, 4th paragraph).

**Reviewer: After removal of the restoration when the replacement was indicated the hardness of dentin was evaluated, please clarify if this was done by the provider or by a different examiner and if the calibration was conducted prior to the assessments.**

 Our response: Dear reviewer, the missing information was provided on Methods section, page 11, and paragraph 1st: “The presence or absence of soft or hard carious tissue is evaluated and recorded by the postgraduate dental student who provides dental care after the restoration removal when replacement is indicated. Training and calibration were conducted before the assessments. An experienced researcher in Cariology performed a theoretical lecture about the clinical characteristics of caries lesions, and training was carried out using photos of clinical cases. The procedure of evaluating the carious tissue is performed to record a possible false-positive diagnosis for dentine caries lesion around the restoration since the authors will also develop an accuracy study nested in this clinical trial”.

**Reviewer: On Adherence, the authors state that “humanized care is provided for all participants. Please provide a definition of “humanized care”.**

 Our response: Dear reviewer, a definition of “humanized care” was provided on the “Adherence” section: “Humanized care is provided for all participants, focusing on the patient's well-being and providing empathy, affection, and familiarity between the CARDEC collaborative group and children and their families”

**Reviewer: Figure 1 provides a very clear flow chart of how lesions were managed according to the ICDAS criteria. A similar chart with the FDI criteria and management would be helpful. Additionally, the predetermined protocols need to be overlaid with the FDI and CARS to better illustrate how the different cases were/are handled.**

 Our response: Dear reviewer, thank you for your suggestion. We created three new flowcharts to illustrate how restorations are managed according to the FDI criteria (Figure 1) and the CARS system (Figure 2). The third flowchart (Figure 3) was done to illustrate how the clinical assessment of restoration is handled differently according to both criteria used in our study. We added on the “Interventions” section of the study protocol the figure numbers after the explanation of the FDI and CARS group. We also added a new sentence to the paper: “A clinical example of the restoration assessment performed with both FDI and CARS criteria is illustrated in Figure 3”.
